# Accuracy and efficiency of an artificial intelligence tool when counting breast mitoses

**DOI:** 10.1186/s13000-020-00995-z

**Published:** 2020-07-04

**Authors:** Liron Pantanowitz, Douglas Hartman, Yan Qi, Eun Yoon Cho, Beomseok Suh, Kyunghyun Paeng, Rajiv Dhir, Pamela Michelow, Scott Hazelhurst, Sang Yong Song, Soo Youn Cho

**Affiliations:** 1 Department of Pathology, University of Pittsburgh Medical Center Cancer Pavilion, Suite 201, 5150 Centre Ave, Pittsburgh, PA 15232 USA; 2 Department of Anatomical Pathology, University of the Witwatersrand and National Health Laboratory Services, Johannesburg, South Africa; 3 School of Medicine, University of Pittsburgh, Pittsburgh, PA USA; 4 Department of Pathology, Samsung Medical Center, Seoul, South Korea; 5 Lunit, Seoul, South Korea; 6 School of Electrical & Information Engineering and Sydney Brenner Institute for Molecular Bioscience, University of the Witwatersrand, Johannesburg, South Africa

**Keywords:** Artificial intelligence, Breast, Carcinoma, Counting, Tumor grade, Digital pathology, Informatics, Mitosis, Whole slide imaging

## Abstract

**Background:**

The mitotic count in breast carcinoma is an important prognostic marker. Unfortunately substantial inter- and intra-laboratory variation exists when pathologists manually count mitotic figures. Artificial intelligence (AI) coupled with whole slide imaging offers a potential solution to this problem. The aim of this study was to accordingly critique an AI tool developed to quantify mitotic figures in whole slide images of invasive breast ductal carcinoma.

**Methods:**

A representative H&E slide from 320 breast invasive ductal carcinoma cases was scanned at 40x magnification. Ten expert pathologists from two academic medical centers labeled mitotic figures in whole slide images to train and validate an AI algorithm to detect and count mitoses. Thereafter, 24 readers of varying expertise were asked to count mitotic figures with and without AI support in 140 high-power fields derived from a separate dataset. Their accuracy and efficiency of performing these tasks were calculated and statistical comparisons performed.

**Results:**

For each experience level the accuracy, precision and sensitivity of counting mitoses by users improved with AI support. There were 21 readers (87.5%) that identified more mitoses using AI support and 13 reviewers (54.2%) that decreased the quantity of falsely flagged mitoses with AI. More time was spent on this task for most participants when not provided with AI support. AI assistance resulted in an overall time savings of 27.8%.

**Conclusions:**

This study demonstrates that pathology end-users were more accurate and efficient at quantifying mitotic figures in digital images of invasive breast carcinoma with the aid of AI. Higher inter-pathologist agreement with AI assistance suggests that such algorithms can also help standardize practice. Not surprisingly, there is much enthusiasm in pathology regarding the prospect of using AI in routine practice to perform mundane tasks such as counting mitoses.

## Background

Handling breast cancer specimens is common in pathology practice. Rendering a pathology report after processing these specimens not only requires an accurate diagnosis, but in the case of invasive carcinoma also requires pathologists to assign the correct histologic tumor grade. A key component of the Nottingham (or modified Scarff-Bloom-Richardson) grading system for invasive breast carcinoma includes the mitotic count [1]. A mitotic count per 10 high-power fields (HPFs) of 0–7 is scored 1, 8–15 is scored 2, and greater than or equal to 16 is given a score of 3. This proliferation activity in breast carcinoma is an important prognostic marker [2]. Some studies have shown that the mitotic count is even a better marker than Ki67 (proliferation index) at selecting patients for certain therapy such as tamoxifen [3].

Counting mitotic figures in hematoxylin and eosin (H&E) stained histology sections is a task typically performed by pathologists while they visually examine a glass slide using a conventional light microscope. Unfortunately, there is substantial inter- and intra-laboratory variation with manual grading of breast cancer in routine pathology practice [4]. This is not surprising, as manually counting mitotic figures by pathologists is subjective and suffers from low reproducibility. Manually counting mitoses can take a pathologist around 5–10 min to perform [5]. Sometimes it may be difficult to discern a mitotic figure from a cell undergoing degeneration, apoptosis or necrosis. There are also differences of opinion on how best to count mitotic figures [6, 7]. The reason for this controversy is that the mitotic activity index depends on the number of mitoses counted in a predefined area (usually in mm^2^) or within a certain number of HPFs that may vary depending on a microscope’s lenses and widefield microscopy view.

Artificial intelligence (AI) coupled with whole slide imaging offers a potential solution to the aforementioned problem. If developed and deployed successfully, an AI-based tool could potentially automate the task of counting mitotic figures in breast carcinoma with better accuracy and efficiency. To date, investigators have validated that making a histopathologic diagnosis in breast specimens can be reliably performed on a whole slide image (WSI) [8]. Moreover, using WSIs to manually count mitoses in breast cancer is reported to be reliable and reproducible [9, 10]. Hanna et al. showed that counting mitotic figures in WSIs outperformed counts using glass slides, albeit this took readers longer using WSI [11]. Several studies have been published showing that digital image analysis can successfully automate the quantification of mitoses [12–18].

Clearly, there is great potential for leveraging digital pathology and AI [19]. AI can benefit pathologists practicing in high, middle and low income countries, especially with the rise in cancer and shortage of anatomical pathologists [20]. However, AI applications in healthcare have not been vigorously validated for reproducibility, generalizability and in the clinical setting [21]. Moreover, hardly any pathology laboratories are currently using AI tools on a routine basis. To the best of our knowledge, there have been no studies addressing whether an AI-based algorithm actually improves pathologist accuracy and efficiency when scoring mitotic figures. The aim of this study was to accordingly critique an AI tool developed to detect and quantify mitotic figures in breast carcinoma.

## Methods

**Fig. 1:**
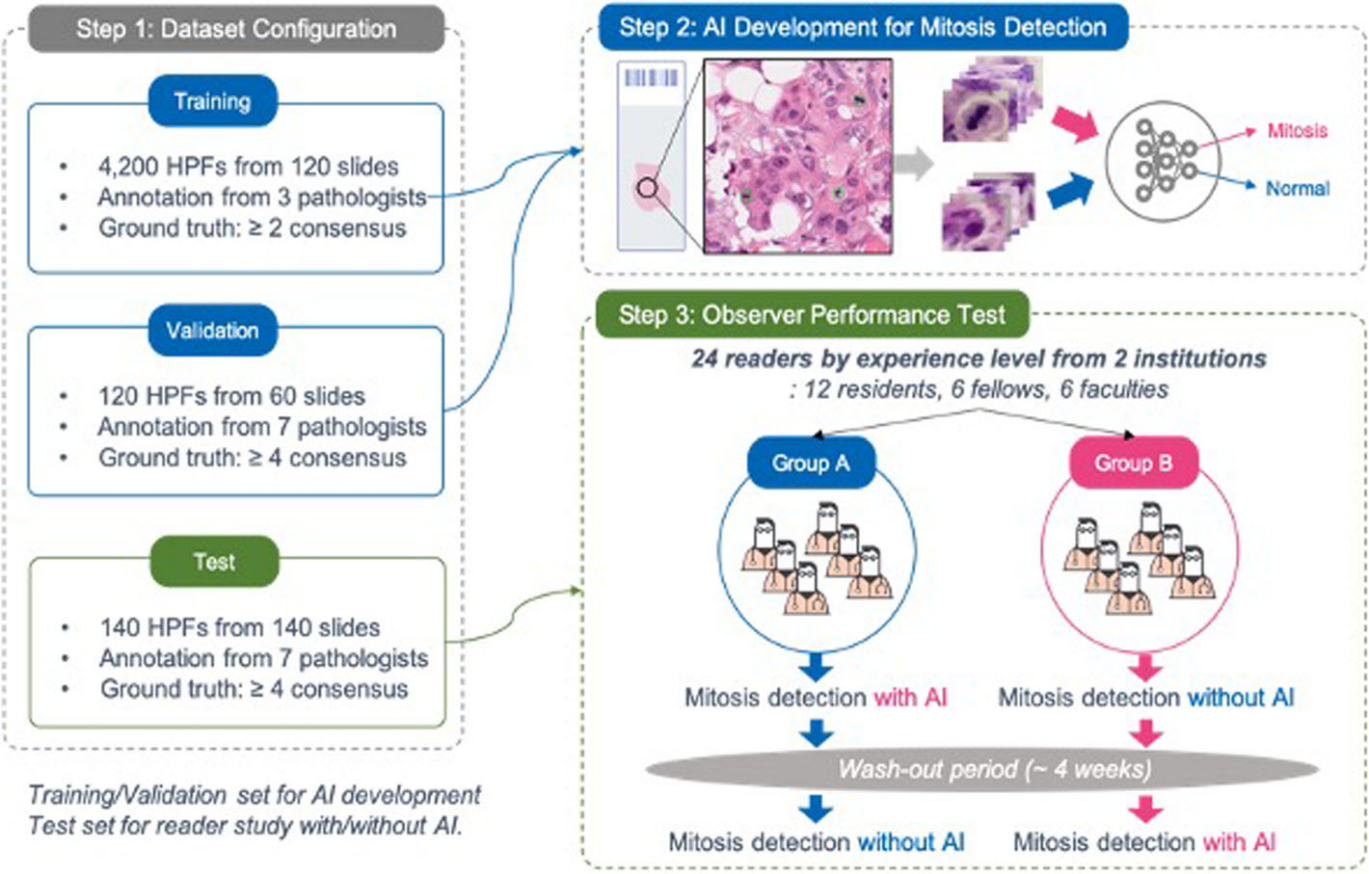
Flow chart of the methodology and datasets employed in developing and validating an AI-based tool to quantify mitoses in breast carcinoma

Figure 1 depicts a flow chart of the methodology and datasets employed in developing and validating the AI-based tool utilized in this study to quantify mitotic figures in digital images of invasive breast carcinoma.

### Datasets

A total of 320 invasive breast ductal carcinoma cases with an equal distribution of grades were selected. Half of these cases were from the archives of the University of Pittsburgh Medical Center (UPMC) in the USA and the rest obtained from Samsung Medical Center (SMC) in Seoul, South Korea. Nearly all of the cases were from females (1 case was from a male with breast cancer). The average patient age was 54.7 years. All cases included were mastectomies with the following range of tumor stages: stage IA (23.6%), IB (7.1%), IIA (31.4%), IIB (23.6%), IIC (0.7%), IIIA (6.4%), IV (0.7%), and data unavailable in 9 cases (6.4%). Table 1 provides a summary of the cancer grade, hormone receptor and HER2 status for enrolled cases (with available data). The average Ki-67 index was 38.3% (Mdn = 34.5%, range 3.0–99.0%). This result was only available in 80 cases, and this subset of cases had higher mitosis scores (*n* = 23 score 2, *n* = 48 score 3) and Nottingham grades (*n* = 34 grade 2, *n* = 41 grade 3). The average proliferation index was accordingly skewed in this subset and higher than would be expected for a typical mixed breast cancer population [22].

**Table 1:** Profile of invasive ductal carcinoma cases enrolled in the study

Reported breast carcinoma parameters		%
Mitosis Score	1	21.4%
	2	31.4%
	3	47.1%
Nottingham Grade	1	7.9%
	2	46.4%
	3	45.7%
ER	Not available	5.0%
	Negative	25.7%
	Positive	69.3%
PR	Not available	5.0%
	Negative	32.1%
	Positive	62.9%
HER2/neu (IHC status)	Not available	5.0%
	Negative	59.3%
	Equivocal	9.3%
	Weakly positive	1.4%
	Positive	25.0%
HER2/neu (FISH status)	Not available	89.3%
	Negative	10.0%
	Positive	0.7%

*ER* estrogen receptor, *FISH* fluorescence in situ hybridization, *HER2* human epidermal growth factor receptor 2, *IHC* immunohistochemistry, *PR* progesterone receptor

A representative H&E glass slide from each case was scanned. At UPMC slides were scanned at 40x magnification (0.25 μm/pixel resolution) using an Aperio AT2 scanner (Leica Biosystems Inc., Buffalo Grove, IL, USA). At SMC slides were digitized at 40x magnification (0.2 μm/pixel resolution) using a 3D Histech P250 instrument (3DHISTECH, Budapest, Hungary). All acquired whole slide image (WSI) files were de-identified. The AI training dataset was comprised of 60 WSIs from UPMC and 60 WSIs from SMC, which provided 16,800 grids (1 grid = ¼ high-power field [HPF]). One HPF is equivalent to 0.19 mm^2^. The AI validation dataset, comprised of another 30 WSIs from UPMC and 30 WSIs from SMC, was used to generate 120 HPFs for annotation. A separate dataset (70 WSIs from UPMC and 70 WSIs from SMC) was subsequently used for a reader study where each WSI file was randomly broken up into 140 representative digital patches (HPFs). Users interacted with individual patches on a computer monitor. The dataset used for analytical validation of the algorithm was different from the dataset selected for the clinical validation study.

### Training (deep learning algorithm)

A deep learning algorithm (Lunit Inc., Seoul, South Korea) was employed for the automated detection of mitoses in digital images [23]. The AI algorithm was trained on an independent dataset, that consisted of 16,800 digital image patches from 120 WSIs (half from UPMC and half from SMC). Three expert pathologists annotated mitoses to construct the ground truth for training. The mitotic figures, which were the consensus of at least two of these pathologists, were used to train the AI algorithm. The algorithm was based on Faster RCNN [24] by ResNet-101 [25] backbone network that has pre-trained weights. The down sampling ratio was 8 and feature maps from the first stage were cropped and resized at 14 × 14 an then max pooled to 7 × 7 for the second stage classifier. Anchor size was 128 × 128 with a single fixed ratio. The number of proposals at the first stage was 2000 to enable a very dense sampling of proposal boxes. Then, box IOU based NMS was performed for post-processing. Various input data augmentation methods such as contrast, brightness, jittering, flip and rotation were performed to build a robust AI algorithm. To select the final model for our reader study, the deep learning algorithm was validated on a separate dataset. Employing the validation dataset we achieved 0.803 mean AP (mAP) which demonstrates good performance. The mAP represents the area under the precision recall curve. A precision recall curve was used to calculate the mAP instead of AUC, because of the large class imbalance (i.e., many non-mitotic cells).

### Ground truth

Seven expert pathologists (4 from UPMC and 3 from SMC) annotated (labeled) mitotic figures in 140 digital image patches using a web-based annotation tool. The tool displayed image patches of breast carcinoma at high magnification, in which clicking on cells automatically generated a square box that annotated the specified cell (i.e. with the mitotic figure present). It required around 10 s to annotate mitotic figures per patch. Pathologist consensus was used to establish ground truth, where agreement of at least 4/7 pathologists was required for each image. Whilst there is no published data available to support the exact number of pathologists required to be in agreement to reach consensus, a consensus of 4 out of 7 was chosen for this study in order to utilize the highest number of cases (*n* = 93, 66.4%) while maintaining consensus among the majority of ground truth makers (57.1%). Table S1 shows the number of cases for each consensus level. Further, for 100% agreement the mitotic figures would likely be very obvious and thus too easy to detect, which would not be suitable to measure performance. Since prior studies have proven that WSI can be used for mitotic cell detection and offers similar reproducibility to the microsocpe [10, 26], we opted to use WSI and not glass slides for establishing the ground truth in this study. Pathologists who annotated slides for ground truth generation did not participate in the subsequent reader study.

### Observer performance test (OPT)

**Fig. 2:**
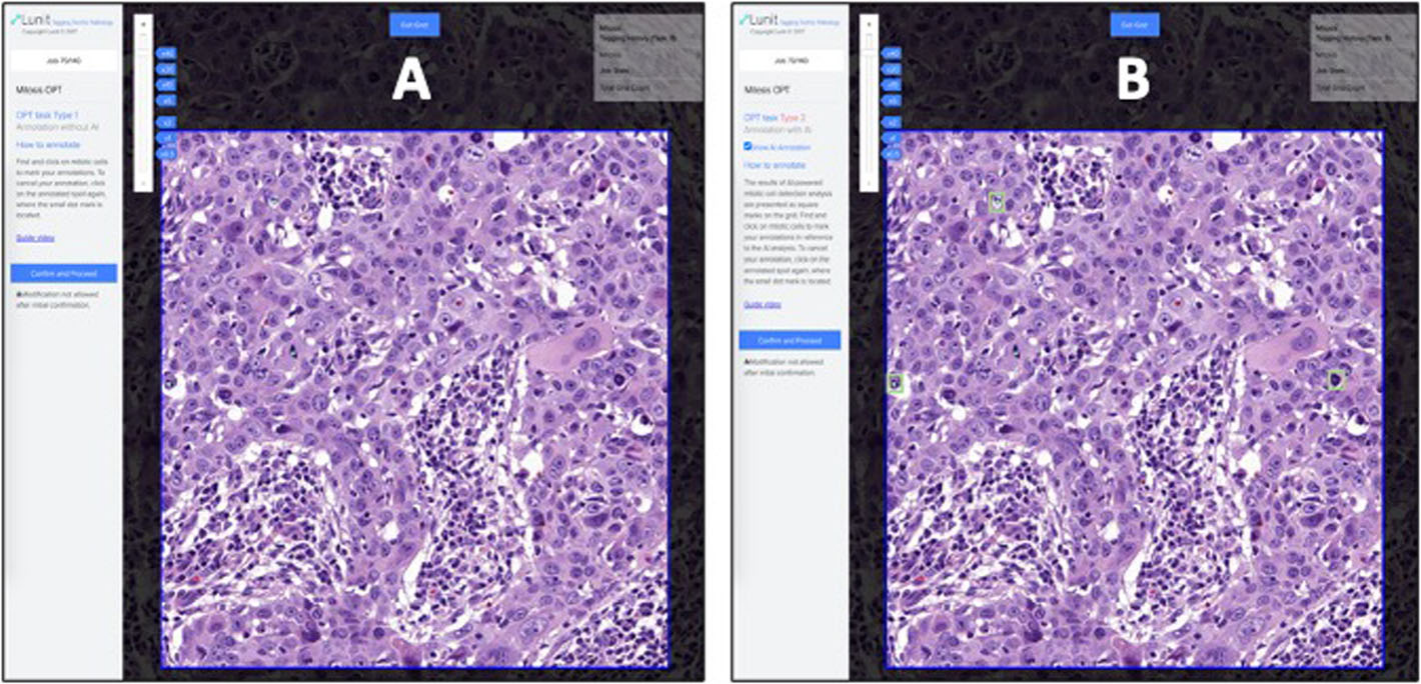
Web-based tool showing a HPF of breast carcinoma. **a** Screenshot of the web-based tool used for the observer performance test without AI. The small green dots indicate mitotic figures marked by the reader. **b** Screenshot of the web-based tool used for the observer performance test with AI. The green boxes indicate mitotic figures detected by AI

For the OPT (reader study), the accuracy and efficiency of mitotic cell detection was compared based on mitotic figure scores provided by humans and the AI algorithm. There were 12 readers at each institution (total of 24 reviewers) that varied in expertise/years of experience (*n* = 6 2nd-4th year pathology residents/registrars, *n* = 3 fellows/post-residency trainees, and *n* = 3 board-certified pathologists). Table S2 summarizes the experience level of all participants involved in the study. Digital slides were presented to test takers in the form of 140 HPFs. Each HPF was equivalent to four digital image patches. There were two reader groups. In group 1 (no AI), readers were first shown HPFs and asked to manually select mitotic figures without AI support. In group 2 (with AI), readers were first shown HPFs where mitotic figures were pre-marked by the AI tool (Figure 2) and asked to accept/reject the algorithm’s selection. Each group repeated this task, but now with/without AI employing a cross-over design to minimize sequential confounding bias. A washout period of 4 weeks was used to control for recall bias between re-reviews of each image. A web-based tool recorded user clicks on images and their time (in seconds) to perform this task. The OPT was replicated at UPMC and SMC institutions. All readers were trained prior to the start of the study, anonymized, and provided informed consent to participate. The readers were not formally asked to provide feedback about their user experience.

### Statistical Analysis

Accuracy of mitotic cell detection was calculated by comparing cells identified by reviewers to cells identified by the ground truth (i.e. consensus of at least 4 of the 7 ground truth makers). Accuracy was compared for reviews with and without AI support for each reviewer. The hypothesis being tested was that reviewer accuracy improves with AI support. To test this hypothesis a Pearson chi-square analysis was performed. For the OPT part of this study, true positive (TP), false positive (FP) and false negative (FN) were calculated with and without AI support. Precision for pathologists was calculated as TP / (TP + FP). Sensitivity was calculated as TP / (TP + FN). As true negatives (TN) represented not only cells, but also all of the white space where no cells were present in an image, TN greatly outnumber the combination of TP + FP + FN and therefore f-scores were calculated (f-score = 2 * ((sensitivity * precision) / (sensitivity + precision)). F-scores closer to 1 indicate perfect detection and precision. Since TN were not calculated, specificity was not possible to calculate.

Efficiency was calculated as seconds spent reviewing each case. The normality of the distribution of the time variable was examined using the Shapiro–Wilk normality test. As the data were not normally distributed, non-parametric statistical tests were used. Wilcoxon signed-rank test was used to compare time spent on the task of counting mitoses with and without AI support. We assumed that image reviews lasting longer than 10 min were outliers (e.g. indicative of an interruption) and thus excluded. Out of the 6720 values in the dataset, 73 (1.1%) were accordingly excluded from analysis. Statistical comparisons were performed for time spent per case with and without AI support for each individual, for each user’s experience level, and overall.

Statistical significance was assumed at *p* < .05. Analysis was performed using IBM SPSS Statistics 22 and Microsoft Excel 365.

## Results

### Accuracy and precision findings

**Fig. 3:**
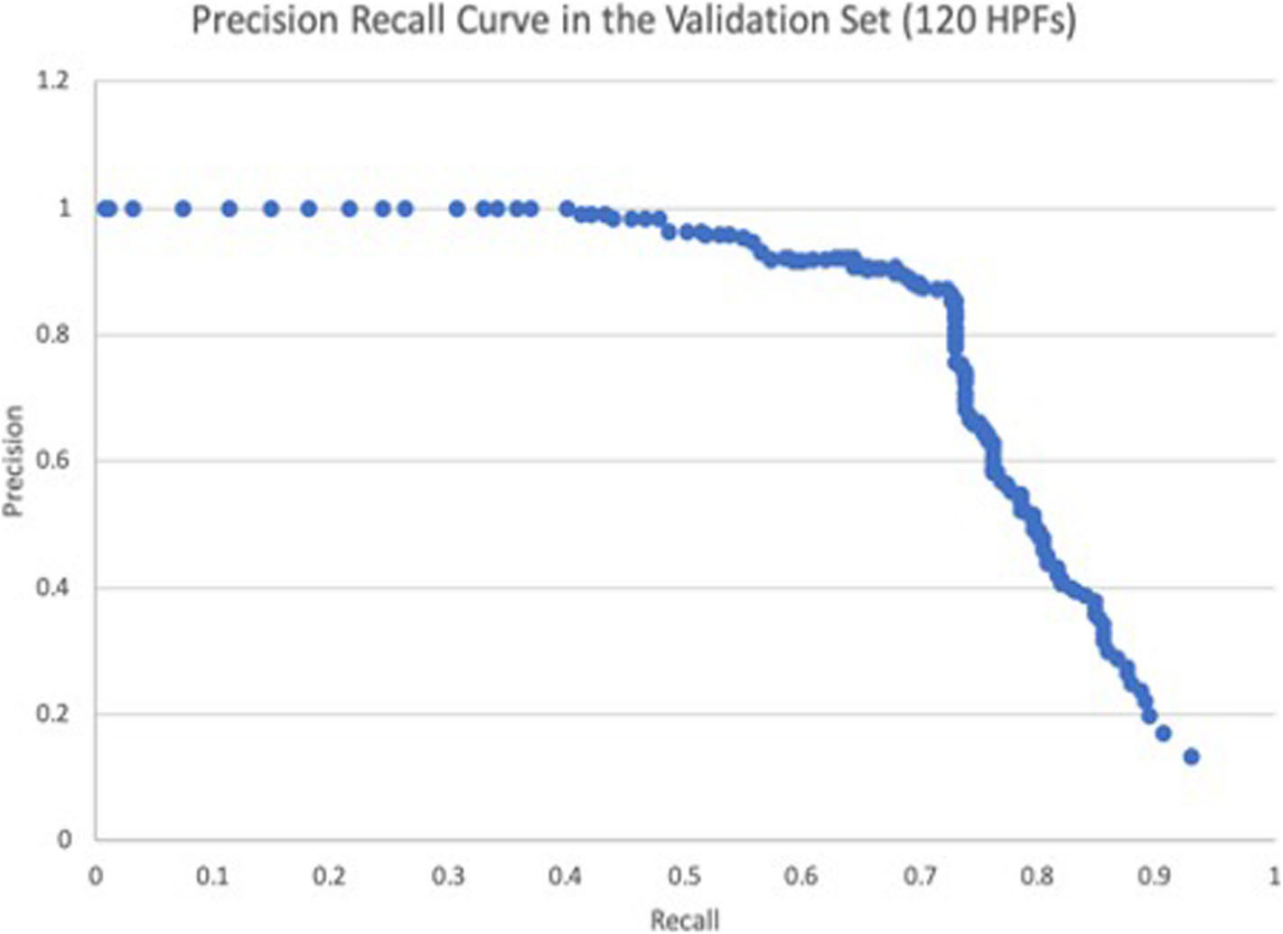
Algorithm performance for mitotic figure detection in the analytical validation dataset

**Fig. 4:**
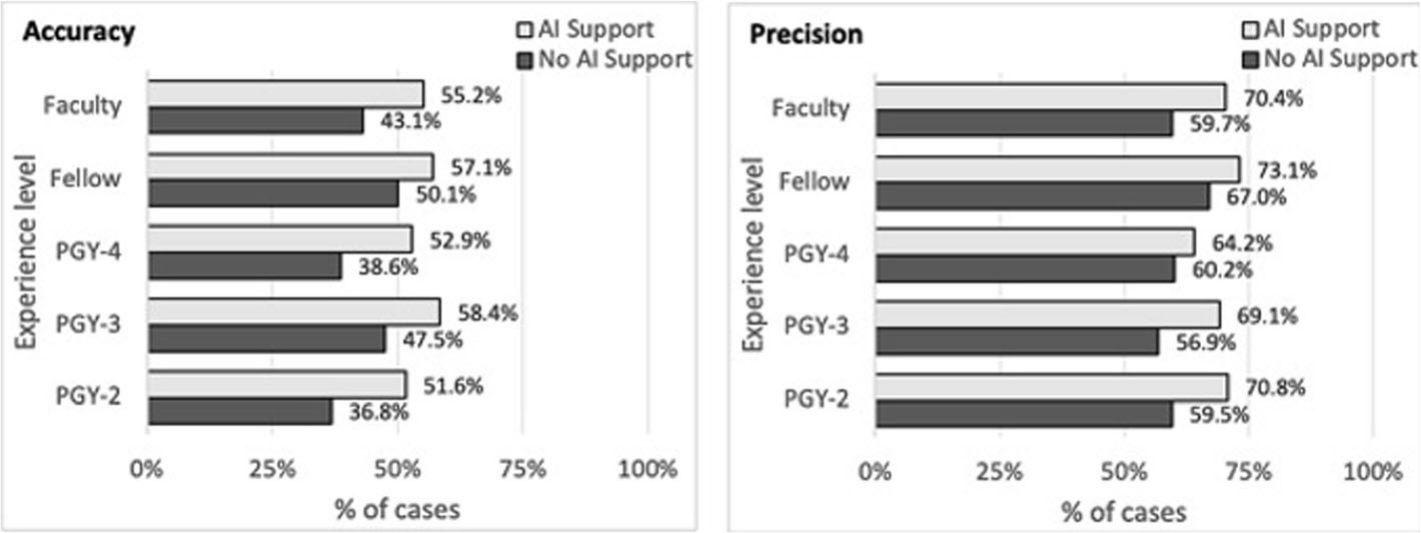
Accuracy and precision with and without AI support per user experience level

A precision recall (PR) curve shows the algorithm’s performance (Figure 3). This PR curve shows the relationship between positive predictive value and sensitivity for every possible cut-off. Akin to the area under a ROC curve (i.e. AUC), the area under the PR curve is large indicating the high recall and precision value of the algorithm at specific cut-offs. Figure 4 shows the accuracy and precision of mitotic cell detection with and without the use of AI support. For each experience level the accuracy and precision were higher with AI support. Table 2 with Chi-square results confirmed that accurate mitotic cell detection was significantly higher with the use of AI support for each experience level. Table S3 shows the individual reviewer accuracy results. Of note, all but one reviewer had higher accuracy with the support of AI. Of the 23 reviewers with improved accuracy, 20 (87%) had a statistically significant increase. Table 3 demonstrates TP, FP and FN values for readers (Table S4 shows individual reviewer results). There were 21 out of the 24 readers (87.5%) that identified more mitoses using AI support. Further, 13 reviewers (54.2%) decreased the quantity of falsely flagged mitoses (FP) using AI support, and 21 (87.5%) decreased the quantity of mitoses that were missed (FN) using AI support. There were six reviewers that falsely detected 100 or more additional mitoses (FP) when screening cases without AI support. Table 3 shows that the number of FPs detected with the use of AI support (2899) is lower than without the use of AI support (3587).

**Table 2:** Accuracy by experience level

User Experience Level	No AI Support	With AI Support	Improved Accuracy with AI support?	*X*^2^ (degrees of freedom)	*p*-value
PGY-2 (*n* = 4)	36.8%	51.6%	Yes	89.30 (1)	**<.001**
PGY-3 (*n* = 4)	47.5%	58.4%	Yes	53.12 (1)	**<.001**
PGY-4 (*n* = 4)	38.6%	52.9%	Yes	87.13 (1)	**<.001**
Fellow (*n* = 6)	50.1%	57.1%	Yes	29.82 (1)	**<.001**
Faculty (*n* = 6)	43.1%	55.2%	Yes	89.84 (1)	**<.001**
**Overall**	**43.9%**	**55.2%**	**Yes**	**320.61 (1)**	**<.001**

*PGY* postgraduate year

**Table 3:** True positive (TP), false positive (FP), and false negative (FN) values for mitotic cell detection

User Experience Level	No AI support			With AI support		
	TP	FP	FN	TP	FP	FN
PGY-2 (*n* = 4)	749	509	779	1003	414	525
PGY-3 (*n* = 4)	1135	861	393	1208	539	320
PGY-4 (*n* = 4)	793	525	735	1149	642	379
Fellow (*n* = 6)	1524	751	768	1659	611	633
Faculty (*n* = 6)	1395	941	897	1647	693	645
**Overall**	**5596**	**3587**	**3572**	**6666**	**2899**	**2502**

*PGY* postgraduate year

Sensitivity for mitotic cell detection increased with the use of AI support for each experience level (Table S5). Sensitivity for mitotic cell detection per individual reviewer was higher for all but 3 reviewers. Precision for mitotic cell detection also increased with the use of AI support for each experience level (Table S6). Sixteen of the 24 reviewers (66.7%) had increased precision with AI support. The f-score (Table S7) for mitotic cell detection without the use of AI support was 0.61, and with the use of AI support was 0.71. The higher f-score with the use of AI suggests that AI support improves overall precision and TP detection of mitotic cells. Cases with AI support also had higher f-scores for each experience level, with 23 of the 24 reviewers (95.8%) demonstrating a higher f-score with AI support. The datasets utilized included only the overall grade (i.e. sum of percent tubules, nuclear pleomorphism and mitoses/10 HPF) for all breast cancers and no details of the exact mitotic figures (i.e. score 1, 2 or 3) for each case. Therefore, we were unable to investigate whether any change in the number of mitoses scored in this study may have altered the grade.

### Efficiency findings

**Fig. 5:**
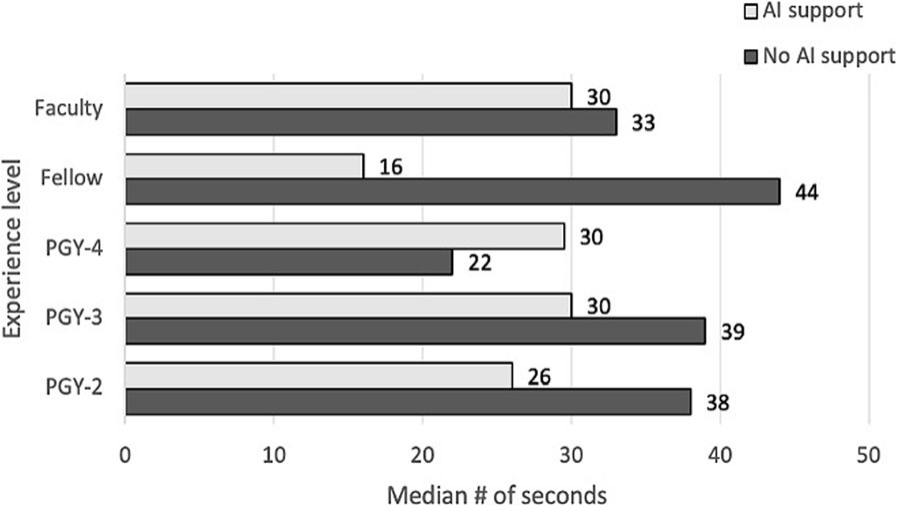
Median number of seconds spent with and without AI support per user experience level

A Wilcoxon signed-rank test indicated that more time was spent on detecting mitotic cells without the use of AI support (median = 36.00 s) than with AI support (median = 26.00 s), Z = − 14.759, *p* < .001, r = .25. Overall, this represents a time savings of 27.8%. Irrespective of whether readers started counting mitoses with or without AI support, nearly all of them read faster with AI assistance, but this was not statistically different. Figure 5 shows the median time spent detecting mitoses with and without AI support by reader experience level. Despite experience level, most participants spent less time detecting mitotic cells with the use of AI support. Fellows had the largest decline, with a median of 44 s spent without the aid of AI compared to 16 s with AI support. The only experience level that had a longer median time spent with AI support was postgraduate year (PGY)-4 users. Table 4 summarizes the median time spent and statistical results per user’s experience level with and without AI support (Table S8 shows individual reviewer results).

**Table 4:** Median time to count mitoses by study participant experience level

User Experience Level	Median # of seconds		AI or no AI faster?	Z	*p*-value	r
	No AI support	With AI support				
PGY-2 (*n* = 4)	38.00	26.00	AI	−8.799	**<.001**	.37
PGY-3 (*n* = 4)	39.00	30.00	AI	−3.290	**.001**	.14
PGY-4 (*n* = 4)	22.00	29.50	No AI	−3.058	**.002**	.13
Fellow (*n* = 6)	44.00	16.00	AI	−16.730	**<.001**	.58
Faculty (*n* = 6)	33.00	30.00	AI	−2.584	**.010**	.09
**Overall**	**36.00**	**26.00**	**AI**	**−14.759**	**<.001**	**.25**

*r* effect size, *PGY* postgraduate year

## Conclusions

There are formidable challenges with successfully translating AI in healthcare [10, 19, 26]. Some of these challenges include technical difficulties, complex implementations, data ownership issues, lack of reimbursement, delayed regulatory approval, ethical concerns, and overcoming human trepidation regarding AI (e.g. mistrust related to the ‘black box’ phenomenon of AI). Bairnov et al. showed that an AI-based decision support tool in Radiology had significant differences with accuracy and inter-operator variability depending on how AI was deployed (i.e. sequential or independent workflow) [21]. To the best of our knowledge, no studies have been published examining the interaction of pathology end users with AI to determine the pros and cons of using AI to assist with counting mitoses. Such studies would provide much needed translational evidence that could help develop recommendations and guidelines for the safe and effective use of AI in routine diagnostic Anatomical Pathology workflow.

This cross validation study demonstrates that pathology end-users were more accurate and efficient at quantifying mitotic figures in digital images of invasive breast carcinoma with the aid of an AI tool that detects mitoses. These data show that the accuracy, sensitivity, precision, and f-scores all increased for each participant experience level with the use of AI support. Readers in both groups had higher inter-pathologist agreement with AI assistance, suggesting that AI can help standardize practice and perhaps result in more reproducible diagnoses. Very few participants unexpectedly had a lower accuracy performance with AI support. The results of this study showed that only 54.2% of reviewers decreased the quantity of falsely flagged mitoses using AI support. The reason why false positives were not reduced across all readers with AI support could be that they missed annotated mitotic figures because they were not clearly visible in the user interface or that some readers may not have believed the AI results. A detailed analysis of the sessions from these individuals showed that for some cases they spent an unusually long time counting mitoses (e.g. 451 s in one case with AI support, but only 15 s on the same case without AI support). This likely points to distraction more than AI causing an actual delay and it is uncertain if these outliers skewed the data. With regard to improved efficiency, the use of AI resulted in a 27.8% decrease in time for mitotic cell detection. In other words, for every 1 h spent searching for cells with mitotic figures without AI support, roughly 16.7 min could be saved using AI support. Nearly every subgroup of participants had faster reading speeds with the use of AI (PGY-4 was the exception). Overall, 66.7% of pathologists read faster with AI (statistically significantly faster for 33.3%). For pathology trainees, use of AI support resulted in faster reads for 83.3% of residents/registrars (statistically significantly faster for 25.0%) and 83.3% of fellows (all 83.3% statistically significantly faster).

Methods to automatically detect mitoses in breast cancer images were introduced in the literature several decades ago [27]. Despite limited access to large digital datasets and prior to the availability of today’s computer processing power, many early image analysis projects demonstrated the feasibility of using computers to assist in counting mitoses [28, 29]. Although some of these first generation algorithms provided promising results, they were not yet suitable for clinical practice. Since then, with the advent of newer technologies including WSI, deep learning methods, graphics processing units and cloud computing we have witnessed a new generation of AI-based algorithms that are able to automate mitosis detection with impressive performance [16, 30–36]. Several international challenges using public datasets catalyzed the development of these sophisticated AI tools [37, 38], including algorithms to predict breast tumor proliferation [39]. The Lunit algorithm utilized in this study to automate mitosis counting in breast carcinoma WSIs integrates three modules: (i) image processing to handle digital slides (e.g. tissue region and patch extraction, region of interest detection, stain normalization), (ii) deep learning mitosis detection network (based on Residual Network or ResNet architecture), and (iii) a proliferation score prediction module [23]. For the Tumor Proliferation Assessment Challenge in 2016 (TUPAC16; http://tupac.tue-image.nl/), Lunit won all tasks including the prediction of mitosis grading. For this specific task their method achieved a Cohen’s kappa score of κ = 0.567, 95% CI [0.464, 0.671] between the predicted scores and the ground truth [17].

In general, mitotic figures are detectable in H&E stained tissue sections due to their hyperchromatic appearance and characteristic shapes. However, it is plausible that mitoses may be missed by humans and/or even AI algorithms due to tissue or imaging artifacts. To address this, using a biomarker such as Phosphorylated Histone H3 (PHH3) may have helped objectively confirm mitotic figures [40, 41]. Even though overall accuracy for readers in the OPT study was determined to be 55.2%, with AI support this was still more sensitive than counting mitotic figures manually. Further, contrary to classifying mitoses into scores 1, 2, and 3 for actual diagnostic purposes, this study was aimed at finding individual mitotic cells in a simulated format, which is expected to have relatively lower performance that could have caused missed or incorrect mitotic figure detection. Davidson et al. have shown that while pathologists’ reproducibility is similar for Nottingham grade using glass slides or WSI, there is still slightly lower intraobserver agreement because grading breast cancer using digital WSI is more challenging [42]. Another limitation of our study was not standardizing the monitors used for annotation and the reader study. However, Norgan et al. showed that manual mitotic figure enumeration by pathologists was not affected by medical-grade versus commercial off-the-shelf displays [43]. In this study we did not equate a glass slide HPF with a digital HPF. Indeed, currently the HPF is typically used in manual microscopy with glass slides when quantifying mitoses (e.g. breast mitoses are evaluated using 10 HPFs at 400x magnification) [44]. However, this HPF at 400x on a glass slide is unlikely to be equivalent to a digital HPF at “40x view” view in a WSI [45].

As verified by this study, expected benefits of adopting AI in pathology practice include automation, elimination of tedious tasks, improved accuracy, and efficiency. Not surprisingly, there is much enthusiasm in pathology regarding the prospect of using AI in routine practice. Interestingly, some of the trainees involved in this study expressed their gratitude for being invited to participate because of the opportunity to experience working with AI first hand. Of course, there is much to still be learned before successfully embedding AI into routine workflows. If AI is indeed more accurate than humans at counting mitoses we will need to determine how this impacts patient outcomes and whether man-made scoring systems may need to be revised.

## Supplementary information

**Additional file 1: Table S1.** Number of cases based on consensus among ground truth makers. **Table S2.** Experience level of participants involved in the OPT component of the study. **Table S3.** Individual accuracy reviewer results for the OPT. **Table S4.** Individual reviewer TP, FP, FN mitotic cell detection results for the OPT. **Table S5.** Sensitivity results by experience level and individual reviewer for the OPT. **Table S6.** Precision results by experience level and individual reviewer for the OPT. **Table S7.** F-scores by experience level and individual reviewer for the OPT. **Table S8.** Individual reviewer results for time spent during the OPT.

## Data Availability

The datasets generated and/or analyzed during this study are not publicly available because they are saved on private servers, but may be available from the corresponding author on reasonable request.

## Abbreviations

AI: Artificial intelligence
AUC: Area under ROC curve
FN: False negative
FP: False positive
H&E: Hematoxylin and eosin
HER2: Receptor tyrosine-protein kinase erbB-2
HPF: High-power field
IBM: International Business Machines Corporation
IOU: Intersection Over Union
mAP: Mean AP (area under precision recall curve)
Mdn: Median
NMS: Non-maximum Suppression
OPT: Observer performance test
PGY: Postgraduate year
PHH3: Phosphorylated Histone H3
PR: Precision recall
RCNN: Regions with convolutional neural networks
SMC: Samsung Medical Center
TP: True positive
TUPAC16: Tumor Proliferation Assessment Challenge in 2016
UPMC: University of Pittsburgh Medical Center
USA: United States of America
WSI: Whole slide image
