# Supplementary material for: Accuracy and efficiency of an artificial intelligence tool when counting breast mitoses
Source: Diagn Pathol. 2020 Jul 4;15:80. doi: 10.1186/s13000-020-00995-z (PMC7335442; doi:10.1186/s13000-020-00995-z)
Supplement: Supplementary file 1 — Additional file 1: Table S1. Number of cases based on consensus among ground truth makers. Table S2. Experience level of participants involved in the OPT component of the study. Table S3. Individual accuracy reviewer results for the OPT. Table S4. Individual reviewer TP, FP, FN mitotic cell detection results for the OPT. Table S5. Sensitivity results by experience level and individual reviewer for the OPT. Table S6. Precision results by experience level and individual reviewer for the OPT. Table S7. F-scores by experience level and individual reviewer for the OPT. Table S8. Individual reviewer results for time spent during the OPT. [file 13000_2020_995_MOESM1_ESM.docx]

**SUPPLEMENTAL TABLES**

**Table S1**. Number of cases based on consensus among ground truth makers.

| Consensus among pathologists | Cases included based on consensus | | Cases excluded based on consensus | |
| --- | --- | --- | --- | --- |
|  | n | % (out of n = 140) | n | % (out of n = 140) |
| 7 out of 7 ground truth makers (100.0%) | 57 | 40.7% | 83 | 59.3% |
| 6 out of 7 ground truth makers (85.7%) | 78 | 55.7% | 62 | 44.3% |
| 5 out of 7 ground truth makers (71.4%) | 87 | 62.1% | 53 | 37.9% |
| 4 out of 7 ground truth makers (57.1%) | 93 | 66.4% | 47 | 33.6% |
| 3 out of 7 ground truth makers (42.9%) | 102 | 72.9% | 38 | 27.1% |
| 2 out of 7 ground truth makers (28.6%) | 112 | 80.0% | 28 | 20.0% |

**Table S2.** Experience level of participants involved in the OPT component of the study.

| Reviewer | Experience | AI support order |
| --- | --- | --- |
| Facility 1, Reviewer A | PGY-2 | AI support second view |
| Facility 1, Reviewer B | PGY-3 | AI support second view |
| Facility 1, Reviewer C | PGY-4 | AI support second view |
| Facility 1, Reviewer D | Fellow | AI support second view |
| Facility 1, Reviewer E | Fellow | AI support second view |
| Facility 1, Reviewer F | Faculty | AI support second view |
| Facility 1, Reviewer G | PGY-2 | AI support first view |
| Facility 1, Reviewer H | PGY-3 | AI support first view |
| Facility 1, Reviewer I | PGY-4 | AI support first view |
| Facility 1, Reviewer J | Fellow | AI support first view |
| Facility 1, Reviewer K | Faculty | AI support first view |
| Facility 1, Reviewer L | Faculty | AI support first view |
| Facility 2, Reviewer A | PGY-2 | AI support second view |
| Facility 2, Reviewer B | PGY-3 | AI support second view |
| Facility 2, Reviewer C | PGY-4 | AI support second view |
| Facility 2, Reviewer D | Fellow | AI support second view |
| Facility 2, Reviewer E | Fellow | AI support second view |
| Facility 2, Reviewer F | Faculty | AI support second view |
| Facility 2, Reviewer G | PGY-2 | AI support first view |
| Facility 2, Reviewer H | PGY-3 | AI support first view |
| Facility 2, Reviewer I | PGY-4 | AI support first view |
| Facility 2, Reviewer J | Fellow | AI support first view |
| Facility 2, Reviewer K | Faculty | AI support first view |
| Facility 2, Reviewer L | Faculty | AI support first view |

PGY = postgraduate year; Facility 1 = Samsung Medical Center, Facility 2 = University of Pittsburgh Medical Center

**Table S3.** Individual accuracy reviewer results for the OPT.

| Reviewers | No AI Support | With AI Support | Improved Accuracy with AI support? | *X*^2^ (degrees of freedom) | p-value |
| --- | --- | --- | --- | --- | --- |
| Facility 1, Reviewer A | 49.4% | 57.5% | Yes | 6.97 (1) | **.008** |
| Facility 1, Reviewer B | 42.9% | 56.0% | Yes | 20.92 (1) | **<.001** |
| Facility 1, Reviewer C | 33.9% | 41.5% | Yes | 9.06 (1) | **.003** |
| Facility 1, Reviewer D | 63.2% | 59.1% | **No** | 1.59 (1) | .207 |
| Facility 1, Reviewer E | 58.4% | 59.8% | Yes | 0.19 (1) | .667 |
| Facility 1, Reviewer F | 51.6% | 51.6% | Yes | 0 (1) | .998 |
| Facility 1, Reviewer G | 46.5% | 57.9% | Yes | 12.80 (1) | **<.001** |
| Facility 1, Reviewer H | 52.8% | 64.5% | Yes | 11.87 (1) | **.001** |
| Facility 1, Reviewer I | 50.6% | 59.3% | Yes | 6.79 (1) | **.009** |
| Facility 1, Reviewer J | 45.1% | 49.3% | Yes | 2.12 (1) | .146 |
| Facility 1, Reviewer K | 43.0% | 57.6% | Yes | 24.29 (1) | **<.001** |
| Facility 1, Reviewer L | 45.6% | 61.3% | Yes | 24.34 (1) | **<.001** |
| Facility 2, Reviewer A | 20.6% | 42.8% | Yes | 61.56 (1) | **<.001** |
| Facility 2, Reviewer B | 52.0% | 61.8% | Yes | 10.75 (1) | **.001** |
| Facility 2, Reviewer C | 39.5% | 54.9% | Yes | 22.20 (1) | **<.001** |
| Facility 2, Reviewer D | 46.0% | 60.7% | Yes | 21.24 (1) | **<.001** |
| Facility 2, Reviewer E | 47.3% | 55.8% | Yes | 7.16 (1) | **.007** |
| Facility 2, Reviewer F | 30.5% | 46.7% | Yes | 36.71 (1) | **<.001** |
| Facility 2, Reviewer G | 32.2% | 47.4% | Yes | 20.83 (1) | **<.001** |
| Facility 2, Reviewer H | 45.4% | 53.1% | Yes | 7.50 (1) | **.006** |
| Facility 2, Reviewer I | 35.2% | 61.8% | Yes | 61.70 (1) | **<.001** |
| Facility 2, Reviewer J | 39.7% | 61.4% | Yes | 39.70 (1) | **<.001** |
| Facility 2, Reviewer K | 44.0% | 56.1% | Yes | 12.63 (1) | **<.001** |
| Facility 2, Reviewer L | 51.6% | 59.4% | Yes | 5.96 (1) | **.015** |

Facility 1 = Samsung Medical Center, Facility 2 = University of Pittsburgh Medical Center

**Table S4.** Individual reviewer TP, FP, FN mitotic cell detection results for the OPT.

| Reviewer | No AI support | | | AI support | | |
| --- | --- | --- | --- | --- | --- | --- |
|  | **TP** | **FP** | **FN** | **TP** | **FP** | **FN** |
| Facility 1, Reviewer A | 250 | 124 | 132 | 318 | 171 | 64 |
| Facility 1, Reviewer B | 312 | 345 | 70 | 293 | 141 | 89 |
| Facility 1, Reviewer C | 276 | 431 | 106 | 291 | 320 | 91 |
| Facility 1, Reviewer D | 331 | 142 | 51 | 246 | 34 | 136 |
| Facility 1, Reviewer E | 277 | 92 | 105 | 296 | 113 | 86 |
| Facility 1, Reviewer F | 231 | 66 | 151 | 230 | 64 | 152 |
| Facility 1, Reviewer G | 242 | 138 | 140 | 270 | 84 | 112 |
| Facility 1, Reviewer H | 214 | 23 | 168 | 282 | 55 | 100 |
| Facility 1, Reviewer I | 208 | 29 | 174 | 283 | 95 | 99 |
| Facility 1, Reviewer J | 261 | 197 | 121 | 306 | 239 | 76 |
| Facility 1, Reviewer K | 277 | 262 | 105 | 296 | 132 | 86 |
| Facility 1, Reviewer L | 246 | 157 | 136 | 279 | 73 | 103 |
| Facility 2, Reviewer A | 121 | 206 | 261 | 207 | 102 | 175 |
| Facility 2, Reviewer B | 301 | 197 | 81 | 319 | 134 | 63 |
| Facility 2, Reviewer C | 161 | 26 | 221 | 297 | 159 | 85 |
| Facility 2, Reviewer D | 248 | 157 | 134 | 276 | 73 | 106 |
| Facility 2, Reviewer E | 241 | 127 | 141 | 274 | 109 | 108 |
| Facility 2, Reviewer F | 224 | 352 | 158 | 278 | 213 | 104 |
| Facility 2, Reviewer G | 136 | 41 | 246 | 208 | 57 | 174 |
| Facility 2, Reviewer H | 308 | 296 | 74 | 314 | 209 | 68 |
| Facility 2, Reviewer I | 148 | 39 | 234 | 278 | 68 | 104 |
| Facility 2, Reviewer J | 166 | 36 | 216 | 261 | 43 | 121 |
| Facility 2, Reviewer K | 180 | 27 | 202 | 258 | 78 | 124 |
| Facility 2, Reviewer L | 237 | 77 | 145 | 306 | 133 | 76 |

Facility 1 = Samsung Medical Center, Facility 2 = University of Pittsburgh Medical Center

**Table S5.** Sensitivity results by experience level and individual reviewer for the OPT.

|  |  | No AI Support | AI Support | Improved Sensitivity with AI support? |
| --- | --- | --- | --- | --- |
| Experience level | PGY-2 | 49.0% | 65.6% | Yes |
|  | PGY-3 | 74.3% | 79.1% | Yes |
|  | PGY-4 | 51.9% | 75.2% | Yes |
|  | Fellow | 66.5% | 72.4% | Yes |
|  | Faculty | 60.9% | 71.9% | Yes |
| Reviewer | Facility 1, Reviewer A | 65.4% | 83.2% | Yes |
|  | Facility 1, Reviewer B | 81.7% | 76.7% | No |
|  | Facility 1, Reviewer C | 72.3% | 76.2% | Yes |
|  | Facility 1, Reviewer D | 86.6% | 64.4% | No |
|  | Facility 1, Reviewer E | 72.5% | 77.5% | Yes |
|  | Facility 1, Reviewer F | 60.5% | 60.2% | No |
|  | Facility 1, Reviewer G | 63.4% | 70.7% | Yes |
|  | Facility 1, Reviewer H | 56.0% | 73.8% | Yes |
|  | Facility 1, Reviewer I | 54.5% | 74.1% | Yes |
|  | Facility 1, Reviewer J | 68.3% | 80.1% | Yes |
|  | Facility 1, Reviewer K | 72.5% | 77.5% | Yes |
|  | Facility 1, Reviewer L | 64.4% | 73.0% | Yes |
|  | Facility 2, Reviewer A | 31.7% | 54.2% | Yes |
|  | Facility 2, Reviewer B | 78.8% | 83.5% | Yes |
|  | Facility 2, Reviewer C | 42.1% | 77.7% | Yes |
|  | Facility 2, Reviewer D | 64.9% | 72.3% | Yes |
|  | Facility 2, Reviewer E | 63.1% | 71.7% | Yes |
|  | Facility 2, Reviewer F | 58.6% | 72.8% | Yes |
|  | Facility 2, Reviewer G | 35.6% | 54.5% | Yes |
|  | Facility 2, Reviewer H | 80.6% | 82.2% | Yes |
|  | Facility 2, Reviewer I | 38.7% | 72.8% | Yes |
|  | Facility 2, Reviewer J | 43.5% | 68.3% | Yes |
|  | Facility 2, Reviewer K | 47.1% | 67.5% | Yes |
|  | Facility 2, Reviewer L | 62.0% | 80.1% | Yes |
| **Overall** | | **61.0%** | **72.7%** | **Yes** |

PGY = postgraduate year; Facility 1 = Samsung Medical Center, Facility 2 = University of Pittsburgh Medical Center

**Table S6.** Precision results by experience level and individual reviewer for the OPT.

|  |  | No AI Support | AI Support | Improved Precision with AI support? |
| --- | --- | --- | --- | --- |
| Experience level | PGY-2 | 59.5% | 70.8% | Yes |
|  | PGY-3 | 56.9% | 69.1% | Yes |
|  | PGY-4 | 60.2% | 64.2% | Yes |
|  | Fellow | 67.0% | 73.1% | Yes |
|  | Faculty | 59.7% | 70.4% | Yes |
| Reviewer | Facility 1, Reviewer A | 66.8% | 65.0% | No |
|  | Facility 1, Reviewer B | 47.5% | 67.5% | Yes |
|  | Facility 1, Reviewer C | 39.0% | 47.6% | Yes |
|  | Facility 1, Reviewer D | 70.0% | 87.9% | Yes |
|  | Facility 1, Reviewer E | 75.1% | 72.4% | No |
|  | Facility 1, Reviewer F | 77.8% | 78.2% | Yes |
|  | Facility 1, Reviewer G | 63.7% | 76.3% | Yes |
|  | Facility 1, Reviewer H | 90.3% | 83.7% | No |
|  | Facility 1, Reviewer I | 87.8% | 74.9% | No |
|  | Facility 1, Reviewer J | 57.0% | 56.1% | No |
|  | Facility 1, Reviewer K | 51.4% | 69.2% | Yes |
|  | Facility 1, Reviewer L | 61.0% | 79.3% | Yes |
|  | Facility 2, Reviewer A | 37.0% | 67.0% | Yes |
|  | Facility 2, Reviewer B | 60.4% | 70.4% | Yes |
|  | Facility 2, Reviewer C | 86.1% | 65.1% | No |
|  | Facility 2, Reviewer D | 61.2% | 79.1% | Yes |
|  | Facility 2, Reviewer E | 65.5% | 71.5% | Yes |
|  | Facility 2, Reviewer F | 38.9% | 56.6% | Yes |
|  | Facility 2, Reviewer G | 76.8% | 78.5% | Yes |
|  | Facility 2, Reviewer H | 51.0% | 60.0% | Yes |
|  | Facility 2, Reviewer I | 79.1% | 80.3% | Yes |
|  | Facility 2, Reviewer J | 82.2% | 85.9% | Yes |
|  | Facility 2, Reviewer K | 87.0% | 76.8% | No |
|  | Facility 2, Reviewer L | 75.5% | 69.7% | No |
| **Overall** | | **60.9%** | **69.7%** | **Yes** |

PGY = postgraduate year; Facility 1 = Samsung Medical Center, Facility 2 = University of Pittsburgh Medical Center

**Table S7.** F-scores by experience level and individual reviewer for the OPT.

|  |  | No AI Support | AI Support | Improved f-score with AI support? |
| --- | --- | --- | --- | --- |
| Experience level | PGY-2 | 0.54 | 0.68 | Yes |
|  | PGY-3 | 0.64 | 0.74 | Yes |
|  | PGY-4 | 0.56 | 0.69 | Yes |
|  | Fellow | 0.67 | 0.73 | Yes |
|  | Faculty | 0.60 | 0.71 | Yes |
| Reviewer | Facility 1, Reviewer A | 0.66 | 0.73 | Yes |
|  | Facility 1, Reviewer B | 0.60 | 0.72 | Yes |
|  | Facility 1, Reviewer C | 0.51 | 0.59 | Yes |
|  | Facility 1, Reviewer D | 0.77 | 0.74 | No |
|  | Facility 1, Reviewer E | 0.74 | 0.75 | Yes |
|  | Facility 1, Reviewer F | 0.68 | 0.68 | Yes |
|  | Facility 1, Reviewer G | 0.64 | 0.73 | Yes |
|  | Facility 1, Reviewer H | 0.69 | 0.78 | Yes |
|  | Facility 1, Reviewer I | 0.67 | 0.74 | Yes |
|  | Facility 1, Reviewer J | 0.62 | 0.66 | Yes |
|  | Facility 1, Reviewer K | 0.60 | 0.73 | Yes |
|  | Facility 1, Reviewer L | 0.63 | 0.76 | Yes |
|  | Facility 2, Reviewer A | 0.34 | 0.60 | Yes |
|  | Facility 2, Reviewer B | 0.68 | 0.76 | Yes |
|  | Facility 2, Reviewer C | 0.57 | 0.71 | Yes |
|  | Facility 2, Reviewer D | 0.63 | 0.76 | Yes |
|  | Facility 2, Reviewer E | 0.64 | 0.72 | Yes |
|  | Facility 2, Reviewer F | 0.47 | 0.64 | Yes |
|  | Facility 2, Reviewer G | 0.49 | 0.64 | Yes |
|  | Facility 2, Reviewer H | 0.62 | 0.69 | Yes |
|  | Facility 2, Reviewer I | 0.52 | 0.76 | Yes |
|  | Facility 2, Reviewer J | 0.57 | 0.76 | Yes |
|  | Facility 2, Reviewer K | 0.61 | 0.72 | Yes |
|  | Facility 2, Reviewer L | 0.68 | 0.75 | Yes |
| **Overall** | | **0.61** | **0.71** | **Yes** |

PGY = postgraduate year; Facility 1 = Samsung Medical Center, Facility 2 = University of Pittsburgh Medical Center

**Table S8.** Individual reviewer results for time spent during the OPT.

| Reviewer | Median # of seconds | | AI or no AI faster? | Z | p-value | r |
| --- | --- | --- | --- | --- | --- | --- |
|  | **No AI support** | **AI support** |  |  |  |  |
| Facility 1, Reviewer A | 27.00 | 24.00 | AI | -0.300 | .764 | .03 |
| Facility 1, Reviewer B | 44.00 | 28.00 | AI | -6.323 | **<.001** | .53 |
| Facility 1, Reviewer C | 52.00 | 47.00 | AI | -0.962 | .336 | .08 |
| Facility 1, Reviewer D | 69.00 | 14.50 | AI | -9.978 | **<.001** | .84 |
| Facility 1, Reviewer E | 22.00 | 15.00 | AI | -6.156 | **<.001** | .52 |
| Facility 1, Reviewer F | 26.00 | 21.00 | AI | -1.714 | .087 | .14 |
| Facility 1, Reviewer G | 42.00 | 38.00 | AI | -1.395 | .163 | .12 |
| Facility 1, Reviewer H | 9.00 | 12.00 | No AI | -4.525 | **<.001** | .38 |
| Facility 1, Reviewer I | 17.50 | 16.00 | AI | -0.124 | .901 | .01 |
| Facility 1, Reviewer J | 37.00 | 45.00 | No AI | -5.576 | **<.001** | .47 |
| Facility 1, Reviewer K | 35.00 | 38.50 | No AI | -1.748 | .080 | .15 |
| Facility 1, Reviewer L | 30.50 | 28.50 | AI | -0.820 | .412 | .07 |
| Facility 2, Reviewer A | 51.00 | 31.50 | AI | -7.046 | **<.001** | .60 |
| Facility 2, Reviewer B | 43.00 | 38.00 | AI | -0.486 | .627 | .04 |
| Facility 2, Reviewer C | 25.00 | 50.00 | No AI | -5.747 | **<.001** | .49 |
| Facility 2, Reviewer D | 64.50 | 12.00 | AI | -10.226 | **<.001** | .86 |
| Facility 2, Reviewer E | 37.00 | 7.00 | AI | -10.004 | **<.001** | .85 |
| Facility 2, Reviewer F | 41.00 | 43.00 | No AI | -0.098 | .922 | .01 |
| Facility 2, Reviewer G | 36.00 | 17.00 | AI | -7.912 | **<.001** | .67 |
| Facility 2, Reviewer H | 76.00 | 53.00 | AI | -1.299 | .194 | .11 |
| Facility 2, Reviewer I | 12.00 | 8.50 | AI | -1.658 | .097 | .14 |
| Facility 2, Reviewer J | 54.50 | 32.00 | AI | -4.269 | **<.001** | .36 |
| Facility 2, Reviewer K | 30.00 | 25.00 | AI | -2.650 | **.008** | .22 |
| Facility 2, Reviewer L | 36.50 | 27.50 | AI | -3.592 | **<.001** | .30 |

r = effect size; Facility 1 = Samsung Medical Center, Facility 2 = University of Pittsburgh Medical Center
